# A novel technique for rapid localization of pulmonary nodules on-site in operating room followed by lung resection: a case series

**DOI:** 10.1097/JS9.0000000000002256

**Published:** 2025-01-28

**Authors:** Wei Huang, Chengcheng Zhang, Weibiao Zeng, Dong Lin, Jiang Fan, Liang Wu

**Affiliations:** Department of Thoracic Surgery, Shanghai General Hospital, Shanghai Jiao Tong University School of Medicine, Shanghai, People's Republic of China

**Keywords:** case series, lung surgery, pulmonary nodules, rapid localization of pulmonary nodules on-site

## Abstract

**Background:**

The localization of pulmonary nodules is crucial for surgical intervention. However, a safe, simple, and efficient method remains elusive. This study aims to evaluate the safety and feasibility of a newly developed preoperative localization method for pulmonary nodules called Rapid Localization of Pulmonary Nodules On-Site (RLPN-OS).

**Methods:**

This study is a single-center, single-arm prospective investigation that collects and analyses the clinical data of patients who underwent RLPN-OS and lung resection, primarily evaluating the safety and feasibility of this technique.

**Results:**

A total of 200 lung nodules from 190 patients who underwent RLPN-OS and partial lobectomy were included in this study. The success rate of localization was 98.0%, and minor intercostal bleeding was observed in 3 (1.5%) cases. All targeted lesions were located and resected successfully. No patients reported experiencing anxiety or pain during or after the procedure.

**Conclusions:**

This novel RLPN-OS technology represents a safe, feasible, patient-friendly, and cost-effective method for lung nodule localization. It has the potential to serve as an alternative to traditional CT-guided percutaneous localization techniques.

## Graphical abstract

**Figure:**
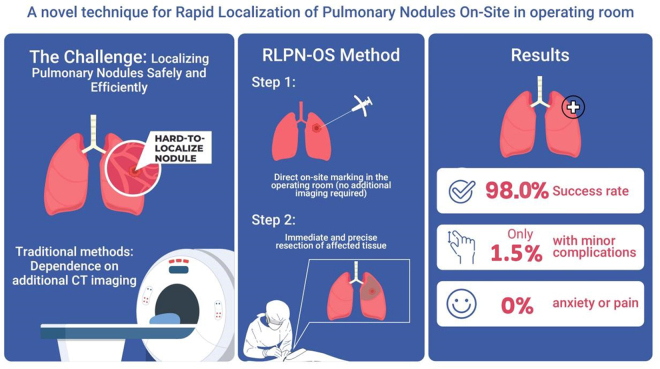

**HIGHLIGHTS**

- This study presents and evaluates a novel technique for the rapid localization of pulmonary nodules on-site in the operating room.
- RLPN-OS is a safe, efficient, and most importantly, patient-friendly method.
- RLPN-OS reduces medical costs by eliminating the need for additional CT scans and extra workforce.

## Background

The application of low-dose computed tomography (CT) in lung cancer screening has been steadily increasing, leading to the detection of more pulmonary ground-glass nodules (GGNs)^[1]^. Currently, video-assisted thoracoscopic surgery is the standard method for resecting suspicious malignant pulmonary nodules. However, determining their precise location for accurate resection can be challenging when pulmonary nodules are too small or too far from the visceral pleura to be seen or palpated^[2]^. Preoperative localization is often required for such nodules that are difficult to locate during intraoperative palpation. Common preoperative localization methods include hookwire^[3,4]^, methylene blue^[5]^, microcoil^[6–9]^, endoscopy^[10]^, and robotic-assisted bronchoscopy^[11]^. However, these methods are associated with limitations. Specifically, hookwire localization may cause anxiety and pain for patients, and there are risks of pneumothorax and bleeding^[3,4]^. Although methylene blue staining is simple and easy to perform, the dye may spread, which can affect the accuracy of localization^[5]^. Microcoil localization requires CT guidance, increasing the patient’s exposure to CT radiation^[6–9]^. Endoscopic localization techniques are technically demanding and may not reach all nodule locations due to equipment limitations^[10]^. Robot-assisted bronchoscopy may increase costs and require specialized operators^[11]^.

We have developed a novel technique called “Rapid Lung Nodule Localization On-Site (RLPN-OS)” to address the current challenges in the preoperative localization of pulmonary nodules. This technology utilizes preoperative chest CT scans to acquire parameters of the patient’s chest anatomy and the location of lung lesions. Without the need for real-time CT scan guidance, the procedure can be performed in the operating room, thus avoiding CT radiation exposure and simplifying surgical operations. The invasive procedure of RLPN-OS, Localization by puncture, is only performed after the patient is under general anesthesia, preventing discomfort after puncture. In addition, this technique only requires reusable measuring tools and cost-effective puncture needles, thereby reducing medical expenses. This study aims to introduce the technical details of RLPN-OS and evaluate its safety and feasibility in patients with pulmonary nodules.

## Methods

### Patient enrollment

This case series has been reported in line with the PROCESS Guideline^[12]^. This study is a single-arm prospective study registered on the Research Registry (registration number: Researchregistry10816). The research adheres to the revised version of the Declaration of Helsinki.

The sample size was determined based on a single-arm objective performance criterion. According to literature data, the success rate of the targeted localization exceeds 96%^[13,14]^, with the lower bound of the 95% confidence interval exceeding 86%. The significance level (*α*) was set at 0.025, and the statistical power (1 − *β*) is 80%. Using these parameters, the required sample size was calculated to be *n* = 72.

$$n=\frac{{\left[Z_{1-\frac{\alpha }{2}}\sqrt{P_{0}\left(1-P_{0}\right)}+Z_{1-\beta }\sqrt{P_{T}\left(1-P_{T}\right)}\right]}^{2}}{{\left(P_{T}-P_{0}\right)}^{2}}$$

In this formula, *n* denotes the sample size, *P*₀ represents the target value, *P_T_* is the anticipated efficacy value, *α* signifies the Type I error, and *β* indicates the Type II error. This study included patients who underwent lung nodule resection surgery at Shanghai General Hospital between September 2021 and November 2024. All surgeries were performed by thoracic surgeons with more than 10 years of surgical experience. The main inclusion criteria were as follows: (1) the presence of suspected malignant mixed nodules or pure ground-glass opacity (pGGO)^[15]^; (2) a solitary pulmonary nodule with a maximum diameter of less than 20 mm; and (3) in cases where multiple nodules were found on the same side, nodules suspected of malignancy and smaller than 8 mm were resected together with the primary lesion. The exclusion criteria were as follows: (1) nodules located near the mediastinum or major heart vessels, posing a high risk of vascular injury; and (2) patients with significant obesity (BMI >28) and unclear surface anatomical landmarks on the chest, making it difficult to palpate the ribs and determine the body surface marker points of the nodules. The criteria for successful localization in this study were as follows: (1) the distance between the lung surface marker and the nodule did not exceed 20 mm, measured as the shortest straight-line distance from the edge of the lesion to the anchoring claw; and (2) based on the lung surface marker, the pulmonary nodule was successfully resected and located. The primary endpoints of this study were the safety and success rate of localization surgery, with particular attention to complications such as intercostal bleeding, pulmonary vascular bleeding, and pulmonary or air embolism.

### Detailed stjpg for RLPN-OS

**Figure 1.**
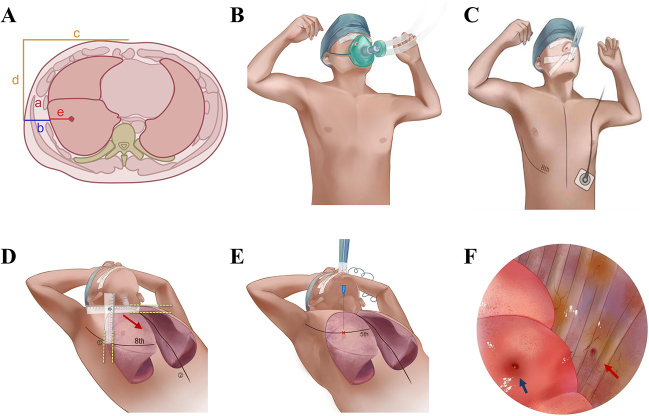
Flow diagram illustrating the detailed stjpg of RLPN-OS. (A) Four parameters are acquired from thin-slice chest CT images to determine the localization point of a lung nodule projected on the skin surface. (a) Intercostal position (intercostal space where the target lung nodule is located); (b) chest wall thickness; (c) horizontal distance from the midline of the sternum; (d) vertical distance from the midline of the sternum. (B) Measurement of the patient’s tidal volume during deep breathing. (C) General anesthesia and double-lumen endotracheal intubation were administered and airway pressure was measured at the end of inspiration. (D) Determining the body surface localization point: utilizing a specially designed ruler consisting of horizontal and vertical components, the surface projection location of the nodule is determined based on four parameters obtained from preoperative CT scans, and this location is marked as the surface localization point. The localization ruler moves in the direction of the arrow. (E) Simulate lung expansion and puncture to generate lung surface markers. Use a puncture needle to puncture the skin vertically from a marked point on the surface of the body. The red arrow point to body surface marker points. (F) Locate lung surface markers for lesion resection. The red arrow points to the pinhole left by the puncture needle in the inner chest wall, and the blue arrow points to the pinhole left by the puncture on the lung surface, that is, the lung surface marking point.

**Figure 2.**
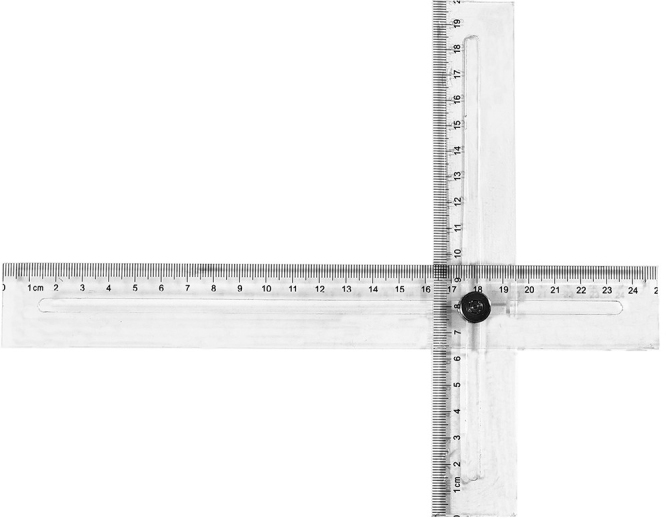
Localization ruler used for RLPN-OS. The localization ruler mainly consists of two right-angle rulers with lengths of 20 and 25 cm, respectively. These two rulers are fixed together with a metal rivet, and a slide rail is left in the center of the rulers for the rivet to move. The graduation of the ruler can be adjusted by moving the position of the rivet based on the chest anatomical parameters obtained from preoperative CT scans.

**Step 1:** Acquire nodule localization parameters from preoperative CT and adjust the scale of the localization ruler. The location of the target pulmonary nodule was evaluated using thin-slice chest CT (slice thickness: 0.625 mm). Four parameters were obtained from the thin-slice chest CT to determine the location of the pulmonary nodule on the skin surface (Fig. 1A): (a) intercostal space position; (b) chest wall thickness; (c) horizontal distance from the sternal midline; (d) vertical distance from the sternal midline. Then the scale of the localization ruler was adjusted based on the values of parameters c and d. The localization ruler is a specialized measurement tool consisting of horizontal and vertical components (as shown in Fig. 2).

**Step 2:** Airway pressure measurement and general anesthesia (Fig. 1B, C): The purpose of measuring airway pressure is to simulate the lung expansion state during CT scanning, ensuring that the degree of lung expansion during puncture localization is consistent with that during CT scanning, thereby minimizing localization errors. The specific procedure is as follows: First, the is positioned supine on the operating bed, with both hands placed behind the head, maintaining the same position as during the CT scan. The anesthesiologist puts an oxygen mask on the patient, instructs them to take a deep breath and hold it, records the tidal volume during deep breathing, and calculates the average of three measurements. Next, the anesthesiologist administered general anesthesia and double-lumen endotracheal intubation to the patient, initiated mechanical ventilation with the previously calculated average tidal volume using an anesthesia machine, and measured the airway pressure at the end of inhalation, which corresponds to the pressure during the CT scan.

**Step 3:** Determine the body surface localization point (Fig. 1D): To determine the surface localization point: (1) Locate the patient’s sternum, draw the midline of the sternum, and count the ribs to identify the intercostal space corresponding to the nodule. (2) Align the horizontal component of the scaled localization ruler with the marked sternal midline and place the vertical component close to the upper edge of the ribs near the nodule. (3) Move the vertical ruler horizontally toward the pulmonary nodule.

The surface localization point of the nodule is the intersection of the lower end of the vertical component of the localization ruler and the marked line on the upper edge of the rib.

**Step 4:** Simulated lung expansion and puncture to generate lung surface localization point (Fig. 1E): The anesthesiologist performs manual ventilation to maintain the airway pressure at the previously measured level, simulating the expanded state of the lungs at the end of inhalation during the CT scan. At this point, a puncture needle (e.g. the needle from a 20-mm syringe) is inserted perpendicularly into the target lung area through the body surface localization point, creating tiny bleeding points on the lung surface as localization points.

**Step 5:** Locate the lung surface localization point for lesion resection (Fig. 1F). After entering the thoracic cavity, locate the markers left by the puncture needle on the lung surface. Determine the resection scope based on these markers and proceed with lung resection.

Videos 1 (http://links.lww.com/JS9/D798) and 2 (http://links.lww.com/JS9/D797) in the Supplementary Digital Content demonstrate the detailed procedure of RLPN-OS performed in a case of lung nodule.

For pulmonary nodules located below the mid-axillary line, the patient’s position should be changed to lateral decubitus during RLPN-OS localization, while all other procedures remain unchanged. In cases where pleural adhesions are suspected, a small amount of methylene blue can be injected onto the lung surface using a puncture needle (e.g. a 20-mm syringe) as a marker to prevent difficulty in finding the marker due to bleeding during adhesion separation.

### Statistical analyses

All statistical analyses were performed using SPSS version 22. Quantitative data are presented as medians with interquartile ranges (IQRs), and categorical variables are described using frequencies and percentages. Continuous variables were analyzed using the two-tailed Student’s *t*-test, and categorical variables were analyzed using the *χ*^2^ test or Fisher’s exact test, as appropriate. A *P*-value of <0.05 was considered statistically significant.

## Results

### Patient and nodules characteristics

A total of 200 nodules from 190 patients were included in this study, with 10 (5.3%) patients presenting with multiple nodules. The study cohort consisted of 82 (43.2%) males and 108 (56.8%) females, with a median age of 54 years (IQR, 44.8–65 years). Patient and nodule characteristics are summarized in Table 1. Among the nodules, 20 (10.0%) were classified as solid nodules, 36 (18.0%) as mixed GGNs, and 144 (72.0%) as pure GGNs. The median nodule diameter was 7.0 mm (IQR, 5.5–10.0 mm). Nodule locations were distributed as follows: 70 (35.0%) in the right upper lobe, 11 (5.5%) in the right middle lobe, 46 (23.0%) in the right lower lobe, 44 (22.0%) in the left upper lobe, and 29 (14.5%) in the left lower lobe. All target nodules were successfully resected and identified on the first attempt, Of these, 159 (79.5%) nodules were resected via wedge resection. 17 (8.5%) nodules underwent lobectomy due to the presence of invasive components detected by intraoperative frozen section, ensuring adequate resection margins.

**Table 1:** Characteristics of patient and pulmonary nodules for localization^a^

Characteristics	*n* (%)
Age, years (median, IQR)	54 (44.8–65)
Sex	
Male	82 (43.2)
Female	108 (56.8)
CT characteristics	
Solid	20 (10.0)
Mixed GGN	36 (18.0)
Pure GGN	144 (72.0)
Size, diameter, mm (median, IQR)	7 (5.5–10)
Nodule number	
Single nodule	180 (94.7)
Multiple nodule	10 (5.3)
Nodule location	
Right superior lobe	70 (35.0)
Right middle lobe	11 (5.5)
Right inferior lobe	46 (23.0)
Left superior lobe	44 (22.0)
Left inferior lobe	29 (14.5)
Pathological diagnosis	
Begin	8 (4.0)
AAH	12 (6.0)
AIS	88 (44.0)
MIA	74 (37.0)
IAC	16 (8.0)
Metastatic	2 (1.0)
Resection	
Wedge resection	159 (79.5)
Segmentectomy	24 (12.0)
Lobectomy^b^	17 (8.5)

Data are expressed as *n* (%) unless otherwise indicated.

AAH, atypical adenomatous hyperplasia; AIS, adenocarcinoma in situ; GGN, ground-glass nodule; IAC, invasive adenocarcinoma; IQR, interquartile range; MIA, minimally invasive adenocarcinoma.

^a^ 200 nodules from 190 patients were included.

^b^ Lobectomy was performed after the confirmation of invasive disease by frozen section.

### RLPN-OS localization outcomes

Airway pressure was measured in all patients prior to anesthesia, with a median airway pressure of 21.1 cmH_2_O (IQR, 19.4–25.8 cmH_2_O) during puncture localization. Ten patients had two nodules located in different lobes, and all nodules in these patients were successfully localized and resected. Of the 200 nodules, 196 (98.0%) were successfully localized (Table 2). Among them, 87 (43.5%) localization markers were placed within 1–10 mm from the nodules, 110 (55.0%) had a distance of 10–20 mm from the nodule, and 3 (1.5%) cases had a distance exceeding 20 mm, which was considered as localization failure in this study. These three localization deviations occurred during early attempts. The first case involved a puncture needle that was not perpendicular to the skin. The second case occurred in an obese female patient, where difficulty in determining the rib position led to localization deviation. The third case involved a tall middle-aged male with a nodule in the right lower lobe, where significant respiratory motion and minor airway pressure errors caused the localization point to deviate from the lesion. In addition, one failure was due to the inability to locate the localization marker. This patient had pleural adhesions, and during the separation process, excessive bleeding obscured the localization marker.

**Table 2:** Characteristics of localization procedure (*N* = 200)

Characteristics	*n* (%)
Airway pressure (median, IQR)	21.1 (19.4–25.8)
Distance from marker to nodule	
0–10 mm	87 (43.5)
10–20 mm	110 (55.0)
20–30 mm	3 (1.5)
Successful localization	196 (98.0)
Unsuccessful localization	4 (2.0)
Marker deviation	3 (1.5)
Marker not found	1 (0.5)
Complications	3 (1.5)
Intercostal bleeding	2 (1.0)
Pulmonary vascular bleeding	1 (0.5)
Pneumothorax	0
Air embolism	0

Data are expressed as *n* (%) unless otherwise indicated.

IQR, interquartile range.

In terms of safety, no deaths or serious complications were reported in any of the patients who underwent RLPN-OS, and no anxiety or pain related to the localization process was observed. Minor complications occurred in three patients (1.5%), all of whom experienced intercostal bleeding. No cases of pulmonary vascular bleeding, air embolism, or pneumothorax were observed. The intercostal bleeding was caused by damage to small intercostal blood vessels during puncture, resulting in minimal bleeding that was easily controlled by applying pressure for 2 min. This complication can be avoided by performing the puncture near the superior margin of the rib.

## Discussion

This study included a total of 200 nodules from 190 patients who underwent RLPN-OS localization, achieving a localization success rate of 98.0%. Minor intercostal vessel bleeding occurred in only three cases (1.5%), and no localization-related anxiety or pain was reported. All target lesions were successfully resected. These findings demonstrate that RLPN-OS is a safe and effective method for lung nodule localization. Moreover, this approach holds significant promise for facilitating the surgical resection of lung nodules, offering several key advantages:

### Reduced complications and patient discomfort

RLPN-OS significantly alleviates the pain, discomfort, and complications commonly associated with conventional preoperative localization techniques, such as pneumothorax and hemorrhage. Traditional localization methods frequently used in clinical practice include hook-wire, microcoil, lipiodol, and dye method^[13,16,17]^. While these methods consistently achieve high success rates (exceeding 91% in most studies)^[18–36]^ (as shown in Table 3), they require percutaneous puncture while the patient is conscious, followed by a waiting period of 0.5–2 h. During this time, patients endure the discomfort caused by both the puncture and the presence of a foreign object in the thoracic cavity. In addition, the prolonged waiting period increases the risk of pneumothorax and excessive bleeding.

**Table 3:** Comparison of RLPN-OS and several preoperative lung nodule localization methods in terms of success rates, complications, and costs

Localization technique	Study	Sample size	Success rates	Complications		Required equipment and costs
				Pneumothorax	Hemorrhage	
Hook-wire	Li *et al.* 2015^[18]^	46	93%	37%	21%	Hook-wire and CT scanning, Inexpensive, about 1000 RMB
	Ichinose *et al.* 2013^[19]^	417	100%	68%	10.00%	
	Iguchi *et al.* 2015^[20]^	11	91%	55%	36%	
	Suzuki *et al.* 2014^[21]^	161	98%	38%	35.00%	
	Seo *et al.* 2012^[22]^	174	95%	40%	36%	
	Huang *et al.* 2014^[23]^	41	95%	13%	5.20%	
Microcoil	Liu *et al.* 2014^[24]^	23	100%	9%	18%	Microcoil and CT scanning, Inexpensive, about 1000 RMB
	Finley *et al.* 2015^[6]^	29	93%	14%	0	
	Su *et al.* 2015^[25]^	101	98%	16%	17%	
	Sui *et al.* 2015^[26]^	98	100%	13%	4%	
Methylene blue	Zhou *et al.* 2020^[27]^	129	95%	10%	3%	Methylene blue and CT scanning, Inexpensive, about 800 RMB
	Sun *et al.* 2020^[28]^	47	98%	4%	-	
	Zhang *et al.* 2022^[29]^	32	100%	10%	10%	
Lipiodol	Kawanaka *et al.* 2009^[30]^	107	100%	31%	15%	Lipiodol, CT scanning and detection devices, about 1500 RMB
	Kim *et al.* 2011^[31]^	68	99%	29%	7%	
	Miura *et al.* 2015^[32]^	103	100%	61%	35%	
	Mogi *et al.* 2015^[33]^	56	98%	38%	16%	
ENB	Kuo *et al.* 2019^[34]^	15	100%	7%	20%	Electromagnetic navigation bronchoscopy system, Expensive, about 10 000 RMB
	Zhang *et al.* 2021^[35]^	181	98%	0%	0%	
	Yang *et al.* 2021^[36]^	12	100%	0%	0%	
RAB	Liu *et al.* 2023^[11]^	33	100%	0%	0%	Robot-assisted bronchoscopy system, expensive, about 30 000 RMB
RLPN-OS	-	78	99%	0%	2.60%	Inexpensive needles and free locator ruler for almost 0 cost

ENB, electromagnetic navigation bronchoscopy; RAB, robot-assisted bronchoscopy.

In contrast, RLPN-OS is performed under general anesthesia, ensuring that patients experience no pain or discomfort. Furthermore, surgery is conducted immediately after puncture localization, minimizing the risks of pneumothorax, excessive bleeding, and displacement of localization markers (e.g. needles or spring coils). This makes RLPN-OS particularly suitable for patients who are pain-sensitive or at higher risk of pneumothorax and bleeding.

### Simplified procedure and reduced resource utilization

Conventional preoperative percutaneous localization methods, such as hook-wire, microcoil, and dye techniques, often require multiple CT scans, collaboration with radiology technicians, and access to specialized CT examination rooms. In contrast, RLPN-OS requires only a preoperative CT scan for data acquisition. The localization process itself is completed in the operating room with the assistance of anesthesiologists, eliminating the need for additional CT scans. This reduces patient radiation exposure, simplifies cross-departmental coordination, and optimizes the use of medical resources.

### Cost-effectiveness

Recent advancements have seen the increasing adoption of electromagnetic navigation bronchoscopy and robot-assisted bronchoscopy for lung nodule localization^[37]^. Both techniques achieve high success rates and low complication rates^[11]^ (Table 3). However, these methods rely on expensive equipment, significantly increasing healthcare costs and limiting their availability in many medical facilities.

In contrast, RLPN-OS does not require costly auxiliary devices. The procedure utilizes only inexpensive puncture needles (e.g. 20 mL syringe needles) and reusable positioning rulers, making it highly cost-effective. This affordability ensures broader accessibility and minimal impact on healthcare expenses, further highlighting RLPN-OS as a practical and efficient localization method.

### Limitations

This study does have some limitations. Principal among these is the constrained sample size and the single-center nature of the research, which limit the generalizability of the findings. For instance, there may not be sufficient evidence to determine which patient groups would benefit most from the use of RLPN-OS for preoperative percutaneous lung nodule localization. Furthermore, the absence of a control group and the lack of quantitative metrics render it difficult to ascertain the technology’s superiority in enhancing patient comfort and simplifying the localization process. Hence, conducting future randomized clinical trials with larger sample sizes and more rigorous designs is imperative, necessitating direct comparisons with other methodologies, such as hook-wires, microcoils, and dyes. In addition, due to the ongoing follow-up with these patients, the study currently lacks of long-term post-RLPN-OS follow-up data, including mortality and recurrence rates, which are planned to be incorporated in subsequent research endeavors. Lastly, since RLPN-OS relies on marking the lung surface when achieving the same airway pressure as during CT scanning, deviations between the marked points and the lesions are unavoidable. However, such deviations are minimal and do not hinder the resection of the lesions.

## Conclusion

In summary, this novel RLPN-OS technology represents a safe, feasible, patient-friendly, and cost-effective method for lung nodule localization. It has the potential to serve as an alternative to traditional an alternative to traditional CT-guided percutaneous localization techniques. Larger-scale, multicenter randomized clinical trials are required to further verify the superiority of this technique over traditional localization methods. In addition, it is imperative to develop more advanced localization and puncture tools to reduce the risk of complications arising from preoperative localization.

## Data Availability

Researchers interested in the study data or individual patient data may contact the corresponding author. Data will be shared with bona fide researchers who provide a valid research proposal after the study is completed.

## Research registration unique identifying number (UIN)

Research Registry (Researchregistry10816.).
